# Cryoprotectants-Free Vitrification and Conventional Freezing of Human Spermatozoa: A Comparative Transcript Profiling

**DOI:** 10.3390/ijms23063047

**Published:** 2022-03-11

**Authors:** Mengying Wang, Plamen Todorov, Wanxue Wang, Evgenia Isachenko, Gohar Rahimi, Peter Mallmann, Vladimir Isachenko

**Affiliations:** 1 Department of Obstetrics and Gynaecology, Medical Faculty, Cologne University, 50931 Cologne, Germany; mengyingwang1993@163.com (M.W.); wangwanxuewwx@gmail.com (W.W.); evgenia.isachenko@uk-koeln.de (E.I.); gohar.rahimi@uk-koeln.de (G.R.); peter.mallmann@uk-koeln.de (P.M.); 2 Institute of Biology and Immunology of Reproduction of Bulgarian Academy of Sciences, Tsarigradsko Highway 73A, 1113 Sofia, Bulgaria; plamen.ivf@gmail.com

**Keywords:** human spermatozoa, cryopreservation, freezing, vitrification, differentially expressed genes, transcriptome stability, DEG

## Abstract

Introduction: Spermatozoa cryopreservation is an important technique to preserve fertility for males. This study aimed at exploring the stability of epigenetics information in human spermatozoa, manipulated by two different technologies, freezing and vitrification. Methods: Spermatozoa samples were distributed into three groups: 1. Fresh spermatozoa (control group), 2. Frozen spermatozoa, 3. Vitrified spermatozoa. Epigenetic differences of fresh and cryopreserved spermatozoa were evaluated using high-throughput RNA sequencing. Results: Differentially expressed genes (DEGs) in frozen (1103 genes) and vitrified (333 genes) spermatozoa were evaluated. The bioinformatical analysis identified 8 and 15 significant pathways in groups of frozen and vitrified spermatozoa, respectively. The majority of these pathways are most relevant to immune and infectious diseases. The DEGs of the fertilization process are not detected during vitrification. The freezing process induces more down-regulation of genes and is relevant to apoptosis changes and immune response. Conclusion: Cryopreservation of human spermatozoa is an epigenetically safe method for male fertility preservation. Cryoprotectant-free vitrification can induce more minor biological changes in human spermatozoa, in comparison with conventional freezing.

## 1. Introduction

Spermatozoa cryopreservation is an important technique to protect human spermatozoa of patients receiving Assisted Reproductive Technology (ART), and patients who require to preserve fertility before undergoing surgery, chemotherapy, or radiotherapy [1,2]. Even though the conventional freezing technique of spermatozoa is extensively used in ART, it is generally reported that the freezing–thawing of human spermatozoa adversely damages vitality. Recently, permeable cryoprotectant-free vitrification is an alternative method that has improved post-thaw spermatozoa features compared to conventional freezing [3], with time saving and less labor, making this procedure more straightforward and quicker in application [3]. It presents an explanation in physiological parameters for why cryoprotectant-free vitrification for some human ejaculates is better than conventional slow freezing and vitrification with cryoprotectants. Spermatozoa vitrification is superior to conventional slow freezing, in total motility and progressive motility, Mitochondrial Membrane Potential (MMP), DNA fragmentation, acrosome, morphology, and cytoskeleton [4,5,6].

However, we know little about the functions and mechanisms of transcript profiles in frozen–warmed and fresh human spermatozoa. A transcriptome is a set of transcripts in one cell or one population of cells at a certain status. Transcriptome analysis assists in studying the identification of differentially expressed genes in distinct cell populations. Researchers can also gain a deeper insight into gene boundary identification, variable cleavage, and transcript variation [7]. RNA sequencing via illumina platforms, based on the mechanism of sequencing by synthesis (SBS), offers a wide range of benefits on high throughput and high accuracy out of low sample requirements. This technical method can be a powerful tool for researching RNA transcriptional activity.

It was reported that the correlation of Differential Expression Genes (DEGs) between fertile and infertile spermatozoa by transcriptome analysis, that differences between three groups reveal that spermatozoa RNA has the pivotal potential of acting as markers for fertility evaluation [8]. The study compared the RNA profiles of spermatozoa obtained from asthenozoospermic, normozoospermic, and fertile samples. In addition, it was shown that a medium quality of semen samples, after one freeze–thaw cycle, shared 98% and 39% of its RNAs with ideal (ejaculate) and poor-quality (three freeze–thaw cycles) models, respectively. The results support the view that the population of spermatozoa RNAs varies rapidly in response to insult, during each freeze–thaw cycle [9]. Despite the importance of transcriptome analysis of spermatozoa, and in the recent studies, transcriptome analysis of boar, bull, and other species of spermatozoa, with different freezability, have been explored using RNA-Seq technique [10,11,12], little is known about the association of transcript profiles with human spermatozoa freezability. 

It is widely accepted that mature spermatozoa are both transcriptionally and translationally silent. However, some recent literature indicates that mature spermatozoa can use mitochondrial RNA transcripts for protein translation during the final maturation steps before fertilization [13,14]. In our previous study, we found that some proteins are altered, resulting in higher or lower expression levels of proteins in spermatozoa subjected to vitrification; due to proteomic study only, we can propose that some functional proteins are not altered/modified that were reported to be altered in most of the conventional slow freezing method studies [15]. Like our studies, the increased or decreased alteration of some proteins in cryopreserved spermatozoa has been reported in some studies [16,17,18]. Zhang et al. [19] reported that RNA-Seq of spermatozoa transcriptome confirmed that the cryopreservation procedure compromises the anti-freeze mechanisms of boar spermatozoa by alterations in the expression levels of microRNAs, and found significant overexpression of spermatozoa-related genes compared with fresh spermatozoa [10].

Simultaneously, it was well reported that cryopreservation markedly alters human and boar spermatozoa’s PRM1 and PRM2 expression levels [20,21]. It was demonstrated that differences in spermatozoa transcript expression play an essential role in spermatozoa functions: motility, capacitation, and chromatin condensation [22,23,24].

Unfortunately, no RNA-seq study has been conducted for frozen–warmed human spermatozoa and investigating the molecular changes between slow freezing and vitrification techniques. Therefore, we do not know exactly what happened to DEG transcripts responsible for spermatozoa functions, including motility, capacitation, and fertilization after spermatozoa slow freezing and vitrification, respectively.

In this study, RNA-Seq was performed to compare transcriptome profiles of spermatozoa obtained from humans that were cryopreserved by slow freezing and vitrification (SF and Vi, respectively) and to identify spermatozoa transcripts associated with cryo-damages. 

## 2. Results

### 2.1. Overview Analysis of RNA Sequencing

The overview of transcriptome sequencing, in fresh and frozen–warmed human spermatozoa, is shown in Table 1. A total of 192,180,478, and 171,745,512 and 183,160,670 raw reads, and 183,898,966, and 162,929,510, and 173,830,938 high-quality clean reads were obtained from RNA libraries of fresh and frozen–warmed spermatozoa, respectively (Table 1). However, the unique mapped reads of the reference genome, in fresh and frozen–warmed spermatozoa, were 128,873,987, 104,616,702 and 115,152,448. After aligning the reads of fresh and frozen–warmed spermatozoa to the reference genome, our sequences covered more than 77% of the annotated exonic gene space. 

**Figure 1:**
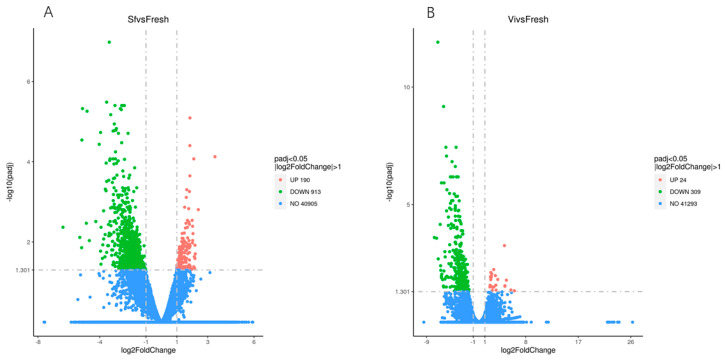
The differential expression genes level in Slow freezing (**A**) and Vitrification (**B**) groups.

Furthermore, 1103 genes were differentially expressed between fresh and slow freezing spermatozoa. From these, 190 genes were up-regulated, and 913 were down-regulated (Figure 1A) [2]. Compared to the fresh spermatozoa, 24 genes were up-regulated, and 309 were downregulated in the vitrification human spermatozoa (Figure 1B). In terms of the overall expression of genes, MEFs had a more comprehensive expression, whereas haEpiSCs shared a similar expression distribution with WT-diploid EpiSCs.

**Figure 2:**
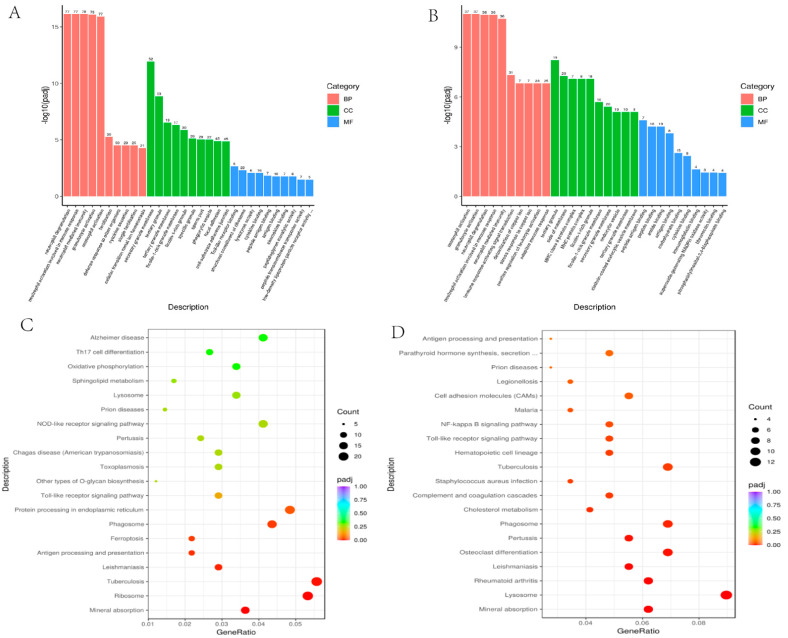
(**A**) Significant Gene ontology terms in Slow freezing group (biological process); (**B**) Significant Gene ontology terms in Vitrification group (biological process); (**C**) Bubble chart of significant KEGG pathways in Slow freezing group; (**D**) Bubble chart of significant KEGG pathway in Vitrification group.

We know that identified genes are common among different treatment groups (Figure 2B), while 926 and 156 differentially expressed genes do not overlap with the fresh spermatozoa. Of these 926 genes, altered only in the slow freezing method, 744 genes were found in low abundance, and 182 were highly abundant (Figure 1A,B); of these 156 genes, only altered in the vitrification group, 140 genes were found in low abundance and 16 in high abundance. Finally, 177 DEGs were significantly differentially expressed among both treatment groups, of which 169 were significantly down-regulated genes, and 8 were up-regulated genes, indicating these genes are susceptible to alteration during the frozen–warmed process used in the study. 

### 2.2. Gene Ontology Enrichment Analysis

Gene Ontology (GO) enrichment analysis was performed to classify DEGs in fresh and frozen–warmed spermatozoa (Table 2). The enriched GO terms were divided into biological process (BP), cellular component (CC) and molecular function (MF) ontologies, shown in Figure 2A,B. After slow freezing, a total of 285 GO terms were significantly annotated and included in three main GO categories. Among the different biological process categories, neutrophil-mediated immunity and granulocyte activation were the most significant and frequently identified enrichment term, followed by defense response to another organism, fertilization, cytokine secretion, and cellular transition metal ion homeostasis. 

Interestingly, within the cellular component category, most transcripts were associated with secretory granule membrane, followed by tertiary granule membrane, ficolin-1-rich granule membrane, phagocytic vesicle, focal adhesion, and cell-substrate adherens junction.

Meanwhile, in the molecular function category, toll-like receptor binding was the most significantly identified enrichment term, followed by a structural constituent of ribosome, lysozyme activity, cytokine binding, peptide antigen binding, chemokine binding, peptidoglycan muralytic activity, peptide transmembrane transporter activity, low-density lipoprotein particle receptor activity. 

When vitrification was compared to the fresh spermatozoa, 412 GO terms were significantly associated with the DEGs; details are depicted in Figure 2. The top ten were greatly enriched in BPs, including neutrophil activation, granulocyte activation, neutrophil degranulation, neutrophil activation involved in immune response, neutrophil-mediated immunity, immune response-activating signal transduction, detoxification of copper ion, the stress response to copper ion, positive regulation of leukocyte activation and adaptive immune response.

For the cell component, the DEGs were enriched in tertiary granule, side of the membrane, MHC class II protein complex, MHC protein complex, ficolin-1-rich granule and membrane, secretory granule membrane, endocytic vesicle, tertiary granule membrane, clathrin-coated endocytic vesicle membrane, and so on. MF analysis showed that the DEGs were significantly enriched in peptide antigen binding, peptide binding, amide binding, antigen binding, carbohydrate binding, cytokine binding, immunoglobulin binding, superoxide-generating NADPH oxidase activity, fibronectin binding, and phosphatidylinositol-3,4-bisphosphate binding.

### 2.3. Kyoto Encyclopedia of Genes and Genomes (KEGG) Pathway Analysis

Kyoto Encyclopedia of Genes and Genomes pathway analysis was used to gain a deeper insight into the pathways of the screened DEGs in this study. As shown in Figure 2C,D, after slow freezing and warming, DEGs were mainly enriched in pathways in Mineral absorption, Ribosome, Tuberculosis, Leishmaniasis, antigen processing and presentation, Ferroptosis, Phagosome, protein processing in the endoplasmic reticulum (Table S1). 

In the comparison between fresh samples and vitrification samples, the identified genes were mainly enriched in mineral absorption, Lysosome, Rheumatoid arthritis, Leishmaniasis, Osteoclast differentiation, Pertussis, Phagosome, cholesterol metabolism, complement, and coagulation cascades, Staphylococcus aureus infection, Tuberculosis, Hematopoietic cell lineage, toll-like receptor signaling pathway, NF-kappa B signaling pathway (Table S2).

### 2.4. Protein–Protein Interaction (PPI) Construction and Hub Gens Identification

**Figure 3:**
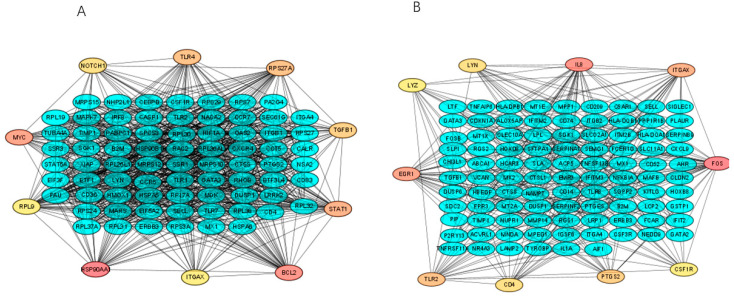
(**A**) Protein–protein interaction in slow freezing group after GSEA enrichment; (**B**) Protein–protein interaction in vitrification group after GSEA enrichment.

Protein interactions among the DEGs were constructed using the STRING database (to ensure the readability of the picture, only display: node degree ≥15). In total, there were 263 nodes and 1727 edges involved in the slow freezing group in the PPI network (Figure 3A), 106 nodes and 336 edges were involved in the vitrification group (Figure 3B). The top 10 nodes were identified according to the degree parameter, and the results showed that HSP90AA1 with the degree = 87 was the most outstanding gene, followed by BCL2 (degree = 68), MYC (degree = 68), STAT1 (degree = 61), RPS27A (degree = 55), TLR4 (degree = 55), TGFB1 (degree = 48), NOTCH1 (degree = 47) and RPL9 (degree = 45), and ITGAX (degree = 45). All of these hub genes were down-regulated in the slow freezing group. The top 10 genes during the vitrification group comprised down-regulated FOS (degree = 36), IL8 (degree = 34), EGR1(degree = 30), ITGAX (degree = 25), TLR2 (degree = 21), PTGS2 (degree = 18), LYN (degree = 17), CD4 (degree = 17), LYZ (degree = 15), and SCF1R (degree = 14). The detailed results of PPI analysis are shown in supplementary Tables S3 and S4.

## 3. Discussion

Spermatozoa cryopreservation is widely used in ART, and we established a methodology of permeable cryoprotectants-free spermatozoa vitrification for human spermatozoa. It is well presented, in some studies, in physiological parameters, why cryoprotectant-free vitrification for human spermatozoa is better than conventional slow freezing and vitrification with the presence of cryoprotectants. Still, no study conducted fresh and frozen human spermatozoa transcriptomics to determine the alterations to transcript profiles during slow freezing and vitrification. In this study, we used RNA-seq to analyze the transcriptome profiles of frozen human spermatozoa. Profiles from humans of the slow freezing and vitrification groups were compared to fresh spermatozoa to identify the relevance of spermatozoa-related transcripts on freezability, responsible for different frozen methods (to investigate the mechanism of spermatozoa cryopreservation at the gene level and provide new options for exploring eventual optimal freezing methods).

### 3.1. Indication from DEGs

In the current study, after the differential expression analysis, it was discovered that vitrification protects spermatozoa genes from alteration. At the same time, the slow freezing method results in more alterations of differential genes, including up-regulation and down-regulation. After using DEseq2 and EdgeR packages, we detected some DEG transcripts commonly expressed in human spermatozoa with both freezing groups. And it is interesting to note that most of the identified DEGs were highly up-regulated in the slow freezing group, compared with the vitrification group. Furthermore, there were only eight transcripts (AC011767.1, GOLGA6L10, ZNF970P, MT-RNR2, GOLGA6L17P, GOLGA6L3, LINC00498, AC091951.1) in high abundance, and most of these genes were golgin A subfamily from unprocessed pseudogenes. Pantano L et al. reported that a dense network of piRNAs was generated from golgin A pseudogenes and targeted their parent protein-coding genes, indicating that pseudogene-derived genes could target and post-transcriptionally regulate the expression of their parent genes in the human male germline. In addition, we also found some processed novel transcripts and antisense pseudogenes up-regulated after slow freezing and vitrification. The finding suggests that the transcript identification and expression evaluation were highly reliable. Even though it is widely accepted that spermatozoa are transcriptionally and translationally quiescent, accumulating evidence has shown that spermatozoa contain a complex RNA population, implicated in male fertility, fertilization, and early embryo development [22,23,25,26].

### 3.2. Findings in GO Terms

After GO enrichment analysis, we discovered that the biological process of a series of neutrophil activities was the most significantly enriched GO term in DEGs, in both groups, indicating that these genes, related to neutrophils, are more easily susceptible crypto-to-crypto during the freezing process. Neutrophils are a key part of the innate immune system and inflammation and provide the first line of defense against infectious agents [27]. Neutrophils work in three main capabilities, in generating oxidative bursts, releasing granules, and forming neutrophil extracellular traps (NETs) [28,29]. They are highly capable phagocytes and have been known to release lytic enzymes and ROS to cleave bacterial resistance factors that affect regeneration cells [30,31]. Hong J. et al. [32] reported that inhibiting the neutrophil response to spermatozoa could block several neutrophil functionalities, such as chemotactic responses, recognition of spermatozoa, signal transduction for a response, or effector functions, such as migration engulfment and phagocytosis. Therefore, the immune response system may be one of the key factors for spermatozoa cryopreservation. In addition, impaired neutrophil functions have the great potential to cause ROS overgeneration ion, which also causes oxidative damage to spermatozoa. While low to moderate levels of ROS are physiological and can be beneficial or necessary for cell survival, high ROS levels damage proteins, DNA, and lipids, taking responsibility for reducing cryopreserved spermatozoa viability. Therefore, we hypothesized that adding the inhibiting agents that block neutrophil activity could counterbalance ROS generation. This way, we might ease the oxidative stress damages to spermatozoa during the freezing process. 

Besides, we identified the differential expressed genes enriched in the top 10 terms in the biological process, the activity of neutrophil activation, granulocyte activation, neutrophil degranulation, neutrophil activation involved in immune response, and neutrophil-mediated immunity was affected in both slow freezing and vitrification groups. However, for neutrophil activation, 37 out of 266 genes were identified during vitrification and all the transcripts were down-regulated during the process. There are 78 out of 826 transcripts identified during slow freezing, and 77 were down-regulated. Thus, slow freezing is more susceptible to the cryo-damage of the neutrophil activation process than the vitrification method. Similarly, among the other biological enrichment processes commonly affected, more related genes were significantly affected in the slow freezing groups than in the vitrification group. Further, the cellular component analysis revealed that identified genes involved in secretory granule membrane, tertiary granule, tertiary granule membrane, ficolin-1-rich granule membrane, damaged during vitrification, are less damaging than slow freezing.

Interestingly, about 30 genes involved in the fertilization process, such as the GLIPR1L1, ADAM30, and SPACA family, were significantly impacted during slow freezing. Still, no genes that participated in the fertilization process were affected during the vitrification process. Our previous study compared protein levels in fresh and vitrified-warmed spermatozoa, using the label-free quantitative proteomic technique. We found similar results that damaged none of the functional proteins responsible for spermatozoa-oocyte interaction. It is well reported that GLIPR1L1 and ADAM play an essential role in the spermatozoa–egg membrane fusion step [33,34]. In this study, we identified that these genes’ responses to the fertilization process were in low abundance after slow freezing. Additionally, Bogle et al. [17] also found that the abundance of SPACA3 decreased in spermatozoa after the conventional freezing method. Still, the SPACA family was not cryo-damaged during our vitrification process, unlike the slow freezing method (SPACA1, SPACA3, SPACA4, SPACA7, SPACA9 were altered). The F-actin capping protein (CAPZ) family assembles and disassembles the outer-acrosomal membrane during capacitation. It was shown that CAPZA3 is decreased in frozen–warmed spermatozoa compared to fresh samples. A higher expression of CAPZB in epididymal spermatozoa, after the conventional slow freezing method, was also reported [35]. Similar to our study, CAPZA3 was also decreased after slow freezing but unaffected in the vitrification. Further, Yamaguchi et al. reported that ODF maintains spermatozoa structures and movement, and the loss of ODF results in non-functional tails and affects spermatozoa motility [36]. It was also confirmed that the expression level of ODF2 was decreased after slow freezing [37]. These results are consistent with our study; we found that ODF3 was down-regulated after the slow freezing method, unaffected in the vitrification process. Our GO enrichment analysis indicated that the spermatozoa vitrification method is more suitable than the traditional slow freezing method.

Moreover, different transcripts were identified in both the cryopreservation process that could affect the genes (RNAs) involved in spermatozoa functionality (including motility, capacitation, acrosome reaction, spermatozoa penetration, and finally fertilization) and early embryonic development. 

### 3.3. Interesting Results from KEGG Pathway Analysis

Kyoto Encyclopedia of Genes and Genomes pathway enrichment analysis of DEGs revealed 8 and 15 significant enrichment pathways in the slow freezing and vitrification groups, respectively. Mineral absorption and Phagosome are common targeted pathways in both groups, indicating high correlation with cryo-damage. We categorized these significantly correlated pathways and found that for slow freezing, most of these genes fall into antigen/protein processing and ribosome, except the common changes; for vitrification, most of the genes fall into two categories that contribute to immune-mediated and human diseases.

### 3.4. What the PPI Analysis Tell Us

In the current study, we performed protein interactions among the DEGs. The ten hub genes selected by PPI were HSP90AA1, BCL2, MYC, STAT1, RPS27A, TLR4, TGFB1, NOTCH1, RPL9, and ITGAX, indicating that these genes may have essential roles during the human spermatozoa slow freezing process; similarly, FOS, IL8, EGR1, ITGAX, TLR2, PTGS2, LYN, CD4, LYZ, CSF1R were selected as the top 10 hub genes in the vitrification group. Moreover, among the top 10 hub genes in the vitrification group, of the three genes (FOS, IL8, EGR1) that exclusively participated in the protein interactions of the vitrification group, the other hub genes were also found involved in the PPI of the slow freezing group, with the higher connectivity degree. However, none of the 10 hub genes in the slow freezing group (except ITGAX) were detected in the vitrification group. Further, our finding showed that the HSP90AA1 and BCL2 had the highest degree in the PPI works, that all were down-regulated and confirmed in the slow freezing method. The connectivity degree was higher in the slow freezing group than in the vitrification group. Higher numbers of nodes were involved in the PPI network in the slow freezing group than the vitrification group. Our results seem to reflect a problem: more altered transcripts were found engaged in the slow freezing method, indicating that slow freezing stimulus has more cryo-damage potential than vitrification.

Notably, FOS is an inflammatory and apoptosis-related gene [38]. The FOS gene family consists of four members, c-FOS, FOSB, FRA-1, and FRA-2, which encode the leucine zipper proteins that dimerize with proteins of the JUN family to form transcription factor activator protein (AP-1), which has multiple functions in the testes [38]. Furthermore, the AP-1 complex regulates gene expression in response to various stimuli, including cytokines and stress, and has been implicated in the regulation of apoptosis, especially in germ cells [38]. FOS is also a critical transcription regulator of cell proliferation, differentiation, and transformation. A recent study by Fraser et al. [11] showed the up-regulation of FOS in poor freezability ejaculates, indicating that spermatozoa are more susceptible to cryopreservation-induced apoptosis than those from good freezability ejaculates. 

In addition, Elweza et al. [39] reported that washed frozen–warmed bull spermatozoa induced the expression of pro-inflammatory genes (IL1B, IL8, TNFA and NFkB2) and the same group confirmed that the response was mediated via the TLR2/4 signaling pathway [40]. Therefore, freezing stimulates the inflammatory-related pathway and apoptotic-related genes, which cause cryo-damage to spermatozoa. The inflammatory-related genes play a pivotal role in spermatozoa physiology and could facilitate the interaction of spermatozoa with the mucosal immune cells of the female reproductive tract [41]. The apoptosis-like changes are the main factors for the significant decrease in spermatozoa quality parameters, such as spermatozoa motility, viability, and acrosomal integrity [42,43,44]. In the current study, we also detected that FOS was exclusively down-regulated during the vitrification process, and the down-regulation of IL-8 and EGR1 was also involved in the vitrification. We hypothesize that the low levels of genes expression of FOS IL8 in the vitrification process could trigger an apoptotic-like mechanism in human spermatozoa during cryopreservation, which could likely result in increased cryo-damage.

HSP90AA1 is a molecular chaperone protein, involved in the folding, transport and assembly of proteins [45,46] localized to spermatozoa flagellum [47]. Heat shock proteins (HSPs) help maintain structure and metabolic processes, providing cells with stress resistance. HSPs comprise a group of unrelated proteins that are classified into the HSP100 (HSPH), HSP90 (HSPC), HSP70 (HSPA), HSP60 (HSPD), and HSP27 (HSPB) families. HSP90AA1 plays essential roles in several cell processes: thermal shock, apoptosis, scavenging for reactive oxygen species (ROS), and spermatozoa motility. Some conflicting reports showed that HSP90AA1 concentrations in boars were increased or decreased after post-thaw [48,49]. However, in humans [50] and bulls [51], levels decreased following cryopreservation. One reason might be that the cell could not produce HSPs because of freezing and protein degradation. Decreases in HSPs preceded decreased motility in cooled and frozen spermatozoa [49,52]. HSP90AA1 is also involved in ATP metabolism. Therefore, it is observed that the common decreased motility of post-thaw spermatozoa may also be due to decreased HSPs, leading to limited ATP availability [51]. In our study, HSP90AA1 was significantly decreased and more involved in the PPI network in slow-frozen samples compared to fresh spermatozoa. Further, we observed a down-regulation of HSPA5, HSPA6, DNAJC3, DNAJC5B, And DNAJB11 after slow freezing. The HSP70 (HSPA) family members seem to be rich components on the surface of spermatozoa [53,54]. it was reported that the down-regulation of HSP70 in spermatozoa was cryopreserved, following the slow freezing method [51]. In addition, in terms of DNAJ (HSP 40), a chaperone protein that prevents protein misfolding in a cell, during our previous study, it was identified that DNAJC3, DNAJC5B, and DNAJB11 were all unaffected during the vitrification process. Therefore, the results of this study are consistent with our previous studies. None of the HSPs were identified as decreased following the vitrification method, unlike the slow freezing process. HSPs were not susceptible to damage during the vitrification method.

*BCL2*, 26 kd in molecular weight and a crucial antiapoptotic factor [55], *Bcl-2* is one of the essential H in the mitochondria-mediated intrinsic apoptotic pathway [56]. It has been established that apoptosis is executed via various proteins, which *Bcl-2* family members regulate [57]. Bcl-2 is the most critical anti-apoptotic protein, and its function is related to interfering with mitochondrial apoptosis pathways [58]. The primary role of the BCL-2 is to prevent the action of pro-apoptotic proteins responsible for pore formation in the mitochondria [59]. Moreover, the supplementation of anti-apoptotic Bcl-2 protein can inhibit the apoptotic pathways [57,60], and it has been confirmed that Bcl-2 protein supplementation exerts its protective effect on spermatozoa, against apoptosis-like changes developed during cryopreservation [61].

In addition, it was also reported the decreased expression level of *Bcl-2* [62] was after spermatozoa freezing; this is similar to the results of our study. We found the gene expression levels of BCL2 were significantly decreased in the slow freezing process but unaffected in vitrification. Therefore, our results suggest that vitrification ameliorates the effects of apoptosis-like changes in human spermatozoa, slow freezing promoting more apoptosis damage to spermatozoa because of the down-regulation of BCL2. 

## 4. Materials and Methods

**Figure 4:**
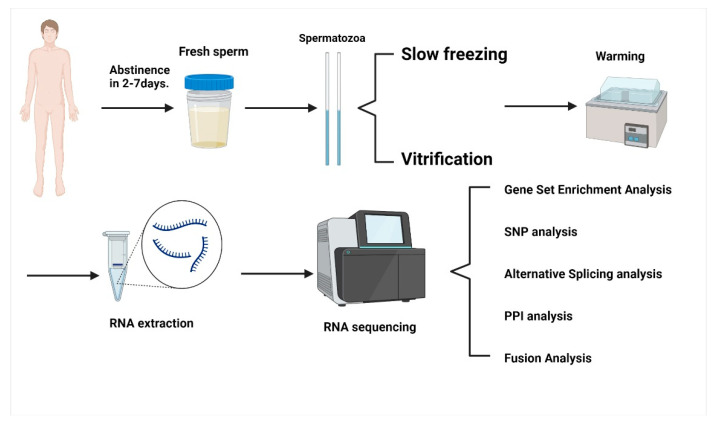
Flow chart of experimental methodology.

The brief description of methodology is shown in Figure 4.

### 4.1. Sample Collection

Semen samples were selected from fertile men by masturbation and ejaculation after at least 48 h of sexual abstinence, who gave informed consent, and this was approved by the Ethics Boards of the University of Cologne. Four fresh ejaculate semen samples with suitable semen quality parameters were selected. After liquefaction, a semen analysis was performed in the reproductive medicine laboratory; all the parameters were evaluated according to published guidelines of the World Health Organization. The manipulations were performed on normozoospermic samples, which means only samples in this study whose physiological parameters progressive motility ≥ 50%, concentration ≥ 15 × 10^6^/mL, pH, and morphology were within the reference values provided by WHO guideline were collected.

### 4.2. Spermatozoa Cryopreservation

All investigations were carried out on spermatozoa prepared by the swim-up technique. The basic medium is Sydney IVF Gamete buffer (Cook Medical, Eight Mile Plains, QLD, Australia) with 10% Dextran Serum Supplement (DSS, Irvine Scientific, Santa Ana, CA, USA). After the swim-up technique, the collected pellet was resuspended in the basic medium to achieve a concentration of 100 × 10^6^ spermatozoa/mL and finally aliquoted into 3 equal sub-samples for the following groups. We cryopreserved 1 mL of spermatozoa suspension (10 straws, each with 100 μL of suspension per sample, were used for conventional freezing with cryoprotectants, and 50 capillaries were used for cryoprotectant-free vitrification).

#### 4.2.1. Spermatozoa Slow Freezing

The collected spermatozoa pellet was diluted in a 1:2 ratio with the freezing medium (12% (*v/v*) glycerol and 20% (*v/v*) egg yolk; Freezing Medium TYB, Irvine Sci.). After that, 100 μL of the spermatozoa suspension was aspirated into the capillaries placed in 0.25-mL plastic straws (MTG GmbH, Bruckberg, Germany). Then, the straws with capillaries were placed horizontally in liquid nitrogen vapor (−160 °C, 10 cm over the liquid nitrogen surface), kept for 30 min, and finally put into the liquid nitrogen. Thawing of the frozen spermatozoa was performed by placing the straws at room temperature. One ml of Sperm Preparation Medium was added to every ten μL of the warmed sample, and the spermatozoa suspension was centrifuged at 380× *g* for 5 min. The supernatant was removed, and the pellet was resuspended in basal medium. Then, an evaluation of the spermatozoa quality was performed.

#### 4.2.2. Spermatozoa Vitrification

As described in our previously published protocol, cryoprotectant-free vitrification was also performed in capillaries [4]. Briefly, ten μL spermatozoa suspension of each treatment group was aspirated in capillaries (Gynemed GmbH & Co. KG, Lensahn, Germany). The capillary was first loaded into 0.25 mL straw (Medical Technology GmbH, Bruckberg, Germany), of which one side was already closed. After sealing the second side, the straw was plunged into liquid nitrogen. For warming, the upper part of the straw was cut, and warming was performed by submerging one by one capillary in 2 mL of a pre-warmed medium at 42 °C for 20 s. Finally, the suspension of spermatozoa was ejected from the capillary for an immediate evaluation of spermatozoa quality.

### 4.3. RNA Extraction and Analysis

The RNeasy Plus Universal Mini Kit was applied to purify the total RNA from 9 human spermatozoa samples. The Sample character of 9 RNA samples was tracked and detected from three aspects: sample quantitation, sample integrity, and sample purity. The RNA sample was applied for library preparation using Illumina compatible kit. Raw data (raw reads) of fastq format were primarily operated through in-house Perl scripts. Total downstream analyses were based on clean data with high quality (for DESeq2 with biological replicates). Differential expression analysis of two conditions/groups (3 biological replicates per condition) was executed using the DESeq2 R package (1.20.0). We used the GSEA analysis tool (http://www.broadinstitute.org/gsea/index.jsp accessed date: 10 February 2022), GO, KEGG, Reactome, DO, and DisGeNET data sets were used for GSEA separately. GATK (v4.1.1.0) software was used to perform SNP calling. rMATS (4.1.0) software was used to analyze the AS event. PPI analysis of differentially expressed genes was based on the STRING database, with known and Predicted Protein-Protein Interactions. We also used Starfusion software (1.9.0) to detect genes that were fused.

## 5. Conclusions

The cryopreservation of human spermatozoa is an epigenetically safe method for male fertility preservation. Cryoprotectant-free vitrification can induce more minor biological changes in human spermatozoa, in comparison with conventional freezing.

## Figures and Tables

**Table 1:** Overview of transcriptome sequencing in fresh and frozen–warmed human spermatozoa.

Group	Raw Reads	Raw Bases	Clean Reads	Clean Bases	GC Content %	%Q30	%Q20	Total cczzMapped Reads	Unique cczzMapped Reads	Positive Mapped Reads	Negative Mapped Reads
Fresh	192,180,478	28.83G	183,898,966	27.58G	48.35	88.87	94.27	136,571,453 (74.26%)	128,873,987 (70.07%)	64,747,047 (35.2%)	64,126,940 (34.87%)
Slow freezing	171,745,512	25.76G	162,929,510	24.44G	50.1	88.24	93.67	112,130,004 (68.82%)	104,616,702 (64.2%)	52,606,155 (32.28%)	52,010,547 (31.92%)
Vitrification	183,160,670	27.48G	173,830,938	26.07G	47.71	89.22	94.3	121,859,357 (70.1%)	115,152,448 (66.24%)	57,780,422 (33.23%)	57,372,026 (33.0%)

**Table 2:** List of crucial significant Gene Ontology terms (Slow freezing vs. Vitrification).

GO ID	Term	*p*-Value	padj
GO:0043312	neutrophil degranulation	2.69 × 10^−20^	7.53 × 10^−17^
GO:0002283	neutrophil activation involved in immune response	3.95 × 10^−20^	7.53 × 10^−17^
GO:0002446	neutrophil mediated immunity	4.58 × 10^−20^	7.53 × 10^−17^
GO:0036230	granulocyte activation	6.67 × 10^−20^	8.21 × 10^−17^
GO:0042119	neutrophil activation	1.22 × 10^−19^	1.21 × 10^−16^
GO:0009566	fertilization	6.64 × 10^−9^	5.45 × 10^−6^
GO:0098542	defense response to other organism	4.52 × 10^−8^	3.04 × 10^−5^
GO:0050663	cytokine secretion	5.39 × 10^−8^	3.04 × 10^−5^
GO:0007338	single fertilization	5.55 × 10^−8^	3.04 × 10^−5^
GO:0046916	cellular transition metal ion homostasis	1.11 × 10^−7^	5.45 × 10^−5^

## Data Availability

The raw data of RNA-seq can be downloaded at “Sequence read archive” on National Center for Biotechnology Information. BioProject ID: PRJNA814701 (http://www.ncbi.nlm.nih.gov/bioproject/814701 accesssed date: 11 February 2022).

## Supplementary Materials

The following supporting information can be downloaded at: https://www.mdpi.com/article/10.3390/ijms23063047/s1.
