# Supplementary material for: Cryoprotectants-Free Vitrification and Conventional Freezing of Human Spermatozoa: A Comparative Transcript Profiling
Source: Int J Mol Sci. 2022 Mar 11;23(6):3047. doi: 10.3390/ijms23063047 (PMC8956043; doi:10.3390/ijms23063047)
Supplement: Supplementary file 1 [file ijms-23-03047-s001.zip › ijms-1630770-supplementary.pdf]

Supplementary Table S1. The significantly enriched KEGG pathways in slow-freezing process.

KEGG ID	Term	pvalue	padj	Genes
hsa04978	Mineral absorption	1.46E-07	4.22E-05	SLC26A3/ATP1A1/MT1H/SLC40A1/HMOX2/MT1F/VDR/SLC30A1/MT2A/MT1M/ATP1B1/MT1G/SLC31A1/MT1X/HMOX1
hsa03010	Ribosome	1.01E-05	0.001465	MRPS15/RPL26L1/RPL32/MRPS12/RPS24/RPL36AL/RPS27/FAU/RPL30/RPS29/RPL38/RPL9/RPS27A/RPL19/RPL37A/MRPL36/RPS3A/RPL31/MRPL17/MRPS10/RPL7A/RPS7
hsa05152	Tuberculosis	4.63E-05	0.004459	TLR4/IFNGR1/LAMP2/TLR1/CD14/CD209/CTSS/CAMP/STAT1/PLK3/FCGR3B/CD74/VDR/ITGAX/CEBPB/TCIRG1/TLR6/TLR2/TGFB1/CYCS/NFYB/BCL2/FCGR2A
hsa05140	Leishmaniasis	0.000154	0.011159	PTGS2/CYBB/TLR4/IFNGR1/ITGA4/STAT1/FCGR3B/TLR2/TGFB1/ITGB1/ELK1/FCGR2A
hsa04612	Antigen processing and presentation	0.000288	0.016619	CTSS/LGMN/CALR/HSPA5/CD74/HSPA6/NFYB/HSP90AA1/CD4
hsa04216	Ferroptosis	0.000537	0.023952	CYBB/ACSL4/SLC40A1/ACSL5/PCBP1/FTMT/SLC39A8/SAT1/HMOX1
hsa04145	Phagosome	0.00058	0.023952	CYBB/TLR4/TUBA4A/LAMP2/CD14/CD209/CTSS/CALR/FCGR3B/CD36/TCIRG1/TLR6/TLR2/ATP6V1E1/STX7/ITGB1/SEC61G/FCGR2A
hsa04141	Protein processing in endoplasmic reticulum	0.001294	0.046759	HSP90B1/DNAJB11/CALR/HSPA5/LMAN1/SSR1/SSR3/DNAJC3/HSPA6/CKAP4/ERO1B/LMAN2/DERL2/SEC61G/UBE2D1/BCL2/DNAJC5B/XBP1/SSR4/HSP90AA1

ID, Identity document; KEGG, Kyoto Encyclopedia of Genes and Genomes

Supplementary Table S2. The significantly enriched KEGG pathways of DEGs in vitrification process.

KEGG ID	Term	pvalue	padj	Genes
hsa04978	Mineral absorption	8.86E-07	0.000137	MT1H/MT2A/MT1G/MT1F/MT1X/MT1M/MT1E/SLC26A3/SLC40A1
hsa04142	Lysosome	1.31E-06	0.000137	CTSL/CTSS/PSAP/ASAH1/ACP5/LAMP2/SLC11A1/LIPA/NPC2/GM2A/AP1S3 /AP1S2/LAPTM5
hsa05323	Rheumatoid arthritis	1.42E-05	0.000988	CTSL/TLR2/FOS/ITGB2/TNFSF13B/ACP5/TNFRSF11A/IL1A/CXCL8
hsa05140	Leishmaniasis	2.85E-05	0.001491	PTGS2/TLR2/FOS/ITGB2/ITGA4/NFKBIA/IL1A/NCF2
hsa04380	Osteoclast differentiation	0.000122177	0.004819	CSF1R/FOS/ACP5/TYROBP/TNFRSF11A/NFKBIA/IL1A/FOSB/LCP2/NCF2
hsa05133	Pertussis	0.000138344	0.004819	CD14/C1QC/FOS/ITGB2/C1QB/IL1A/CXCL8/SFTPA1
hsa04145	Phagosome	0.000347336	0.01037	CD14/CTSL/CTSS/TLR2/CD209/ITGB2/LAMP2/TUBA3E/NCF2/SFTPA1
hsa04979	Cholesterol metabolism	0.00057338	0.01498	LRP1/ABCA1/CYP27A1/LIPA/NPC2/LPL
hsa04610	Complement and coagulation cascades	0.000977354	0.022696	ITGAX/C1QC/PLAUR/ITGB2/C1QB/C5AR1/SERPINF2
hsa05150	Staphylococcus aureus infection	0.001200831	0.025086	C1QC/ITGB2/C1QB/C5AR1/FPR3
hsa05152	Tuberculosis	0.001361837	0.025086	CD14/ITGAX/FCER1G/CTSS/TLR2/CD209/ITGB2/LAMP2/IL1A/CD74
hsa04640	Hematopoietic cell lineage	0.001440334	0.025086	CD14/CD4/CSF1R/CSF3R/ITGA4/IL1A/KITLG
hsa04620	Toll-like receptor signaling pathway	0.001790943	0.028793	CD14/TLR2/FOS/TLR8/CXCL9/NFKBIA/CXCL8
hsa04064	NF-kappa B signaling pathway	0.002357684	0.035197	CD14/PTGS2/TNFSF13B/LYN/TNFRSF11A/NFKBIA/CXCL8
hsa05144	Malaria	0.002858889	0.039834	LRP1/TLR2/ITGB2/CXCL8/SDC2

ID, Identity document; KEGG, Kyoto Encyclopedia of Genes and Genomes

Supplementary Table S3. Top ten hub genes with higher degree of connectivity (Slow freezing vs. Fresh).

Gene Symbol	Gene Description	Degree
HSP90AA1	heat shock protein 90 alpha family class A member 1	87
BCL2	BCL2 apoptosis regulator	68
MYC	MYC proto-oncogene, bHLH transcription factor	62
STAT1	signal transducer and activator of transcription 1	61
RPS27A	ribosomal protein S27a	55
TLR4	toll like receptor 4	55
TGFB1	transforming growth factor beta 1	48
NOTCH1	notch receptor 1	47
RPL9	ribosomal protein L9	45
ITGAX	integrin subunit alpha X	45

Supplementary Table S4. Top ten hub genes with higher degree of connectivity (Vitrification vs. Fresh).

Gene Symbol	Gene Description	Degree
FOS	Fos proto-oncogene, AP-1 transcription factor subunit	36
IL8	interleukin 8	34
EGR1	early growth response 1	30
ITGAX	integrin subunit alpha X	25
TLR2	toll like receptor 2	21
PTGS2	prostaglandin-endoperoxide synthase 2	18
LYN	LYN proto-oncogene, Src family tyrosine kinase	17
CD4	CD4 molecule	17
LYZ	lysozyme	15
CSF1R	colony stimulating factor 1 receptor	14
